# Recent progress and future directions for expanding gastrointestinal endoscopy in low‐ and middle‐income African nations

**DOI:** 10.1002/jgh3.70026

**Published:** 2024-09-16

**Authors:** Aditya Gaur, Hareesha Rishab Bharadwaj, Priyal Dalal, Khabab Abbasher Hussien Mohamed Ahmed

**Affiliations:** ^1^ School of Medicine University of Central Lancashire Preston UK; ^2^ Faculty of Biology Medicine and Health The University of Manchester Manchester UK; ^3^ Faculty of Medicine University of Khartoum Khartoum Sudan

The burden of gastrointestinal (GI) diseases in low‐ and middle‐income African nations is significant and growing, particularly in regions such as sub‐Saharan Africa (SSA). The prevalence of upper GI conditions, including peptic ulcers and esophageal malignancy‐related dysphagia, is notably high in regions like Senegal.^1^ Lower GI conditions, such as hemorrhoids, infectious colitis, and lower GI cancers, are also becoming increasingly prevalent in other low‐ and middle‐income African nations.^2^ The rising incidence of GI malignancies in low‐ and middle‐income African nations leads to severe complications, diminished quality of life, financial distress, and increased morbidity and mortality. The GLOBACAN report of 2012, devised by the International Agency for Research on Cancer (IARC) denoted that over 40% of GI malignancies originated from developing nations.^3^ A recent Global Burden of Disease (GBD) analysis denoted that a substantial proportion of digestive diseases existed in low‐ and middle‐income nations concentrated in Africa. The African region experienced the highest disability‐adjusted life years (DALYs) due to digestive diseases, with the least improvement over the past decade, with the highest DALY rates observed in nations such as Egypt and the Central African Republic. The same study also denoted that the greatest proportional burden of upper digestive system diseases was in southern SSA.^4^ Future projections indicate that low‐ and middle‐income African nations, especially those in SSA, will experience a 73% increase in GI cancer cases by 2030, with uninvestigated dyspepsia being a significant contributing factor.^5^ Consequently, enhancing the availability and quality of GI endoscopy services is imperative to improve the diagnosis and prognosis of GI conditions in low‐ and middle‐income African nations. To this end, this editorial explores the recent progress and future directions for expanding gastrointestinal endoscopy in these regions, emphasizing the urgent need for advancements in this field.

The establishment and expansion of gastrointestinal endoscopy services in low‐ and middle‐income African nations are met with numerous challenges, foremost among which are financial constraints. These financial limitations make the procurement of essential endoscopic equipment difficult. For instance, despite the substantial gastrointestinal disease burden in Eastern Africa, the region's endoscopy capacity remains critically low, with only 0.12 endoscopists, 0.12 gastroscopes, and 0.09 colonoscopes available per 100 000 inhabitants. This stark deficit in equipment is predominantly attributed to financial challenges.^6^ Furthermore, the shortage of medical doctors significantly impedes the development of endoscopy services. The provision of even basic diagnostic endoscopy services is a substantial challenge, let alone more advanced therapeutic endoscopy. A survey of 87 doctors across Ethiopia, Kenya, Malawi, and Zambia revealed that 63 performed endoscopy, 6 conducted endoscopic retrograde cholangiopancreatography (ERCP), and only 2 performed endoscopic ultrasound (EUS), with the majority of these procedures occurring in private facilities. In Nigeria, a country with over 200 million inhabitants, there are merely 200 endoscopy centers, half of which are located in Lagos, and only one public center consistently offers ERCP services.^6^, ^7^ In countries like Malawi, most clinical work, including diagnostic endoscopy, is carried out by non‐physician clinicians (NPCs), while the few available medical doctors are primarily responsible for managing hospitals and health districts, leaving them with limited capacity to oversee clinical endoscopic procedures. Even when functional endoscopic equipment is available, the scarcity of medical doctors and specialists results in low volumes of both diagnostic and therapeutic endoscopic procedures.^7^ To mitigate these challenges, some low‐ and middle‐income African nations have adopted the use of specially trained nurses to perform GI endoscopy, reducing costs and addressing the growing demand from an aging population. Another significant challenge is the prohibitive cost of endoscopic services to the public. For example, a single esophageal banding with injection sclerotherapy procedure costs approximately 300 United States Dollars (USD), making it inaccessible to the majority of the population.^8^

Despite these challenges, efforts to bridge the gap in GI healthcare provision between high‐income nations (HICs) and low‐ and middle‐income African nations have been evident in recent decades. Collaborative initiatives between HICs and low‐ and middle‐income African nations have spearheaded significant advancements in the provision and expansion of GI endoscopies. Such initiatives have focused on restructuring the current practices of GI endoscopies in low‐ and middle‐income African nations, whilst also emphasizing the improvement in technology and infrastructure. One remarkable instance is the partnership between Portugal and São Tomé and Príncipe (STP), which unfolded over eight missions from 2016 to 2021. Prior to this initiative, STP lacked specialized doctors and nurses in gastroenterology. The project's success was predicated on meticulous planning, encompassing the development of a tailored three‐year gastroenterology curriculum, which focused primarily on basic diagnostic and therapeutic upper and lower endoscopy and proctology. Furthermore, the project emphasized the renovation of the national endoscopy unit to contemporary standards, ensuring a conducive environment for medical procedures. Additionally, the introduction of a telemedicine platform facilitated real‐time consultation and training, enhancing knowledge transfer and skill acquisition among local healthcare practitioners. By 2021, these concerted efforts culminated in the STP team's capacity to independently perform intricate procedures such as endoscopic hemostasis and polypectomies, marking a significant milestone in the enhancement of gastroenterology services within STP. The magnitude of progress was evidenced by the local team's accomplishment of conducting 408 upper endoscopies and 130 therapeutic procedures in 2021 alone, indicative of a substantial and sustainable improvement in GI healthcare provision within the region.^2^

In addition to fostering collaborations, recent endeavors have focused on teaching and education, acknowledging the pivotal role of knowledge transfer in healthcare capacity building. A noteworthy example is the Belgian‐Senegalese inter‐university project, where Belgian healthcare personnel provided vital training to local doctors and nurses, laying the groundwork for the project's success in Senegal. During this initiative, three Senegalese endoscopy nurses and two doctors, fellows in gastroenterology at the University Hospital Le Dantec, underwent intensive training in basic therapeutic procedures, endoscope disinfection, and the evaluation of existing therapeutic and diagnostic GI endoscopy techniques during their respective two‐ and six‐month stays in Belgian university hospitals.^1^ Similarly, another initiative saw selected clinical officers from Malawi participating in an 8‐week focused training program in Germany, where they received comprehensive training in diagnostic and therapeutic upper GI endoscopy. Upon their return to Malawi, the outcomes of 1732 consecutive esophagogastroduodenoscopies (OGDs) performed between September 2001 and August 2010 were analyzed, which revealed favorable outcomes.^6^ This underscores the potential of training and education‐focused exchange programs as a potential to address GI‐focused healthcare needs in resource‐constrained settings.

Additional initiatives have focused on innovative methods to reduce the cost of endoscopic procedures for the public. For instance, variceal hemorrhage from portal hypertension, which carries a high mortality rate in most West African countries, is often exacerbated by the lack of affordable endoscopic banding facilities. At Jos University Teaching Hospital in Nigeria, the Endoscopy Unit has recently started performing esophageal variceal banding and injection sclerotherapy. However, the cost of a single‐use variceal band ligator, approximately USD 300, is prohibitively expensive for most individuals in developing countries. To address this, gastroenterologists at the Endoscopy Unit have devised a cost‐effective alternative. They modify the standard variceal banding technique by cutting size 14 Foley urethral catheters to size and reloading them onto previously used Opti‐vu caps. While not the optimal practice, this adaptation reduces the cost to just $30 per session. This modified technique has significantly increased the uptake of the procedure and improved clinical outcomes.^7^ In addition, the World Gastroenterology Organisation (WGO) has set up training centers in different metropolitan cities across the developing world to tackle the issues of training, education, and endoscopy expansion.^9^

Looking forward, there is an urgent need to focus on developing endoscopic facilities in low‐ and middle‐income African nations. National healthcare departments should establish endoscopic centers near high‐risk populations, ensuring accessibility in both rural and urban regions for timely emergency treatments. These centers must be well‐equipped with diagnostic and therapeutic tools to provide comprehensive GI care. Increased advocacy for greater health spending on gastroenterological conditions and endoscopy can help secure the necessary funding. Furthermore, enhanced collaboration between international and national authorities is essential to devise innovative, resource‐appropriate solutions that can reduce intervention costs and overcome financial barriers to endoscopy. To meet the growing demand, it is crucial to increase the number of trained gastroenterologists. This can be achieved by fostering interest through seminars and workshops for medical trainees at the undergraduate level. Additionally, raising public awareness about the prevention and early treatment of diseases leading to gastrointestinal bleeding, such as hepatitis, peptic ulcer disease, and cancer, is vital. Moreover, a renewed emphasis on retaining gastroenterologists within low‐ and middle‐income African nations and actively tackling the brain drain to HICs is imperative. Primary healthcare providers should play a key role in educating, diagnosing, and referring patients who need endoscopic treatment early, thereby reducing the necessity for emergency interventions. Governments must invest more in public healthcare infrastructure to ensure the widespread availability of essential medical services.

Telemedicine presents a promising solution for enhancing GI practice in Africa. Such platforms enable real‐time consultations, improve access to specialist care, and can be crucial in training and supporting local healthcare providers. Despite evidence of its utilization in some African regions, as discussed previously, there is much to learn. A model worth replicating is the Pacific Gastroenterology Telehealth Seminar (PGTS), which since 2017 has successfully addressed professional isolation and enhanced case‐based learning across Pacific Island Nations. By utilizing telehealth technologies like Zoom and instant messaging apps, PGTS has improved patient outcomes and fostered the development of specialized subgroups for focused medical discussions and tele‐mentoring, demonstrating the potential impact of telemedicine in resource‐limited settings. Telemedicine, therefore, not only bridges the gap in specialist care but also fosters sustainable healthcare development in regions with significant resource limitations.^10^
